# Successful Transvaginal Microwave Ablation of a Heterotopic Cervical Pregnancy. A Case Report

**DOI:** 10.1007/s43032-020-00270-y

**Published:** 2020-07-29

**Authors:** Gabriella Schivardi, Salvatore Alessio Angileri, Giampiero Esposito, Letizia Di Meglio, Valentina Brusati, Anna Maria Ierardi, Gianpaolo Carrafiello, Anna Maria Marconi

**Affiliations:** 1 Department of Obstetrics and Gynecology, San Paolo Hospital Medical School, ASST Santi Paolo e Carlo, Via A di Rudinì 8, 20142 Milan, Italy; 2 Department of Radiology, Fondazione IRCCS Cà Granda Ospedale Maggiore Policlinico, Via F Sforza 35, 20122 Milan, Italy; 3 Department of Health Sciences, University of Milano, Via A di Rudinì 8, 20142 Milan, Italy

**Keywords:** Heterotopic pregnancy, Multiple pregnancy, Assisted reproduction, IVF cycle, Microwave ablation, Case report

## Abstract

Heterotopic cervical pregnancy is a rare and potentially dangerous condition where the challenge is the removal of the ectopic pregnancy while preserving the intrauterine one. We present the case of an otherwise healthy 36-year-old woman with heterotopic cervical pregnancy after an IVF cycle for tubal infertility. At 6.5 weeks after a consultation with gynecologists and interventional radiologists, the patient agreed to undergo microwave ablation of the cervical pregnancy. Monthly ultrasound examination showed the persistence of non homogeneous and vascularized tissue in the cervix. Intrauterine pregnancy continued without complications until 37 weeks when a cesarean section was performed for sudden and profuse bleeding, in the presence of uterine contractions. A healthy baby girl was delivered, and the postoperative course was otherwise uncomplicated. Heterotopic cervical pregnancies are rare, and no consolidated and risk-free treatments are available at the moment. Here, we present a case of heterotopic cervical pregnancy treated successfully and without complications with microwave ablation.

**Electronic supplementary material:**

The online version of this article (10.1007/s43032-020-00270-y) contains supplementary material, which is available to authorized users.

## Introduction

Heterotopic pregnancy (HP) is defined as the simultaneous presence of at least an intrauterine pregnancy (IUP) and at least an extrauterine pregnancy (EP). EP can be tubal, ovarian, cervical, cornual, abdominal, intramural, or cesarean scar pregnancy. The incidence, in spontaneously conceived pregnancies, is estimated to be 1 in 30,000 [1], but due to the increasing use of assisted reproductive techniques, it has now reached 1% [2] as most women with heterotopic pregnancies have a previous history of infertility or tubal disease. Cervical HP is particularly challenging given the rarity of the pathology, the lack of a consensus on treatment, and the high incidence of complications, particularly vaginal bleeding, which can lead to hysterectomy. A recent review which included 37 cases from 1989 to 2018 [3] reports that in 29, aiming at maintaining the IU pregnancy, 26, all treated with surgical excision with/without intra amniotic injection of KCl or methotrexate, resulted in live birth. However, only two women did not present complications at the time of the procedure or during pregnancy.

We present a case report of an early heterotopic cervical pregnancy in which microwave ablation (MWA) was used to interrupt the cervical pregnancy. The IUP pregnancy had a normal course and was delivered at term. To our knowledge, not only is this the first reported case of the successful use of MWA for the treatment of heterotopic cervical pregnancies but also the first described case of MWA treatment of any cervical ectopic pregnancy.

## Case Report

**Fig. 1:**
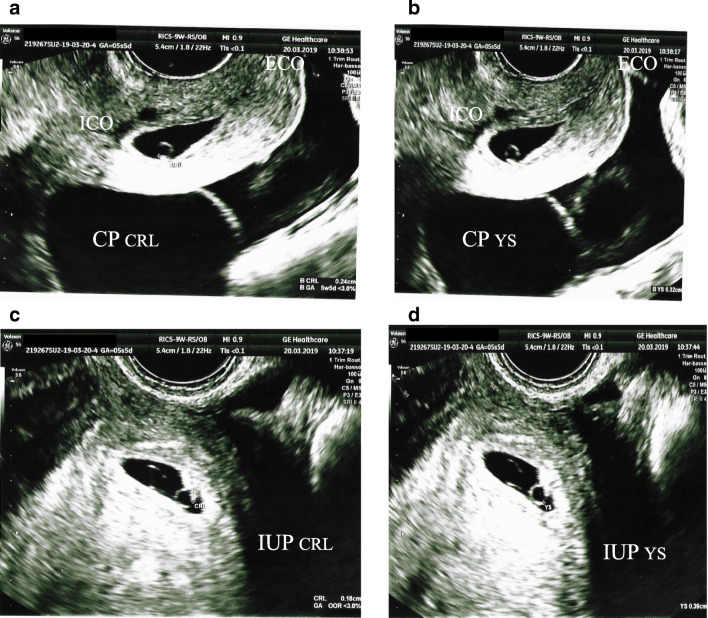
Transvaginal US scan performed at 5^+5^ weeks showing **a** the CRL of 0.18 cm in the cervical pregnancy (CP); ECO, external cervical os; ICO, internal cervical os; **b** the yolk sac (YS) of 0.32 cm in the cervical pregnancy (CP); ECO, external cervical os; ICO, internal cervical os; **c** the CRL of 0.18 cm in the intrauterine pregnancy (IUP); and **d** the yolk sac (YS) of 0.39 cm in the intrauterine pregnancy (IUP)

**Fig. 2:**
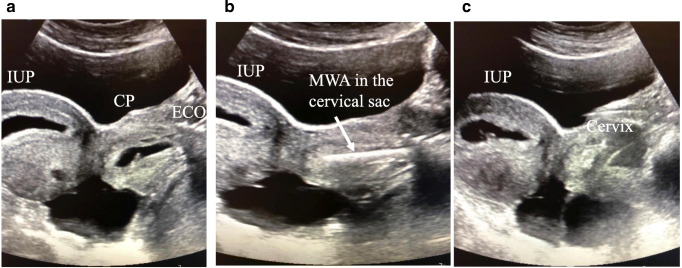
Transabdominal US scan performed at the time of the procedure 6^+5^ weeks: **a** IUP, intrauterine pregnancy; CP, cervical pregnancy; ECO, external cervical os; **b** the microwave antenna (MWA) is inside the cervical sac; and **c** the hyperechoic area as result of the MWA is shown on the right; on the left is the IUP pregnancy

A healthy 36-year-old Caucasian woman gravida 1 was referred to our hospital at 6.3 weeks of gestation due to the presence of a cervical heterotopic pregnancy after an IVF cycle for tubal infertility with transfer of two embryos. She brought an ultrasound exam, performed at 5.5 weeks, showing the presence of two gestational sacs, one intrauterine and one cervical, each with an embryo with a heartbeat and yolk sac (Fig. 1). On admission, transvaginal ultrasound confirmed the presence of two gestational sacs with corresponding embryos and cardiac activity, one located in utero (40 × 21 mm; CRL 6.9 mm) and the other in the cervix (32 × 19 mm; CRL 6.9 mm). On specular examination, the cervical gestational sac was visible in the cervical canal at about 1 cm from the external cervical os, dilated by about 1 cm. No blood loss was present. After an extensive consultation where the woman expressed her intention to do everything possible to safeguard the intrauterine pregnancy, while being aware of the risks related to the termination of cervical pregnancy, and after a multidisciplinary counseling between gynecologists and radiologists, we proposed to use MWA. After written informed consent was obtained, at 6.5 weeks, the patient received one session of MWA. Under transabdominal ultrasound guidance, the microwave antenna was introduced transvaginally inside the cervical gestational sac, and ablation was performed with 100-W output for 90 s (Emprint Ablation System with thermosphere Technology Medtronic; Medtronic, Minneapolis, MN, USA) (Fig. 2). With this MW technology, the output and exposure time are evaluated based on the volume of the target lesion. It was considered that these parameters were the most suitable for obtaining better control and more accurate predictability of the ablated volume, so as to avoid nontargeted ablation. The procedure lasted 15 min overall was performed without anesthesia and with no discomfort for the patient; 1 g cefazolin was given intramuscularly at the same time. The patient did not require any pain medication after the procedure.

**Fig. 3:**
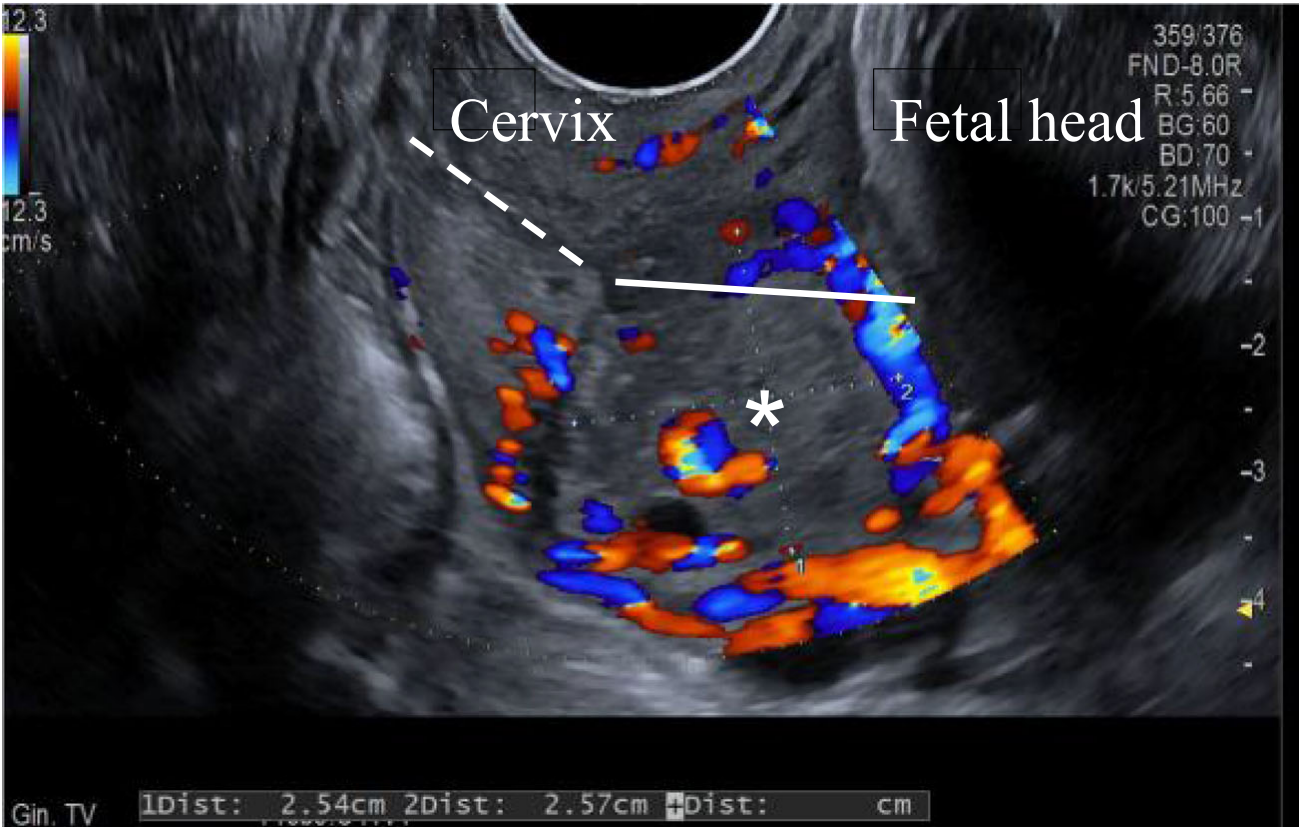
Transvaginal US scan showing the presence [*] within the cervical canal of nonhomogeneous vascularized residual ablation material. The solid and dotted lines represent the two portion of the cervical length

At the end of the procedure, the ablated gestational sac appeared as a homogeneously hyperechoic area, with a mean diameter of 1.8 cm and no flow signal at Color-Doppler US. Neither bleeding nor uterine contractions occurred after the procedure and in the following 3 days, when she was discharged. She was then followed on a regular basis and with monthly obstetric visits and ultrasound examinations that showed the persistence of nonhomogeneous, vascularized material within the cervical canal that was approximately 46 × 42 mm at 15.6 weeks and 25 × 25 mm at 33 weeks (Fig. 3): however, as specular examination and cervical length were always regular, the hypothesis of a cervical cerclage was discarded. Pregnancy was otherwise uneventful.

With an elective cesarean section planned at 39 weeks, at 37.6 weeks, the patient was admitted at the hospital due to abundant vaginal bleeding with irregular uterine contractions, no cervical effacement or dilation present. An emergency cesarean section was performed in the suspicion of placenta abruption, not confirmed neither by the histological examination nor during surgery when it was seen that the blood loss came from the cervico-isthmic area, likely residual of the cervical pregnancy ablation. A live female of 2780 g, Apgar score 10/10, and umbilical arterial pH 7.31 was delivered. A Bakri balloon was placed to prevent further blood loss, which was 800 mL in total. The balloon was removed on the first postoperative day; hemoglobin concentration dropped from 12.2 g/dl before to 9.5 g/dl, 1 day after the cesarean section.

The postpartum course was regular, and the patient was discharged after 4 days, before discharge ultrasound revealed regular endometrium and endocervical canal.

## Discussion

Microwave ablation is largely used to treat solid tumors and has demonstrated to be a precise and safe technique with many potential advantages as it allows to obtain predictable spherical ablation volume, immediate evidence of tissue coagulation on ultrasound, and blood vessels in the coagulation area that do not create ablation zone distortion because of the minimal heat sink effect. The microwaves produced by the system penetrate into targeted tissues causing its coagulative necrosis when the temperature increases [4–6]. In pregnancy, thus far MWA has been used to perform selective feticide in monochorionic diamnotic twin pregnancies [7, 8] including cases of twin reversed arterial perfusion [8, 9] and to treat successfully a rare case of diaphragmatic ectopic pregnancy [10]. All these procedures were performed with a transabdominal approach under US guidance, and most pregnancies were > 20 weeks gestation.

Here, we describe the first case where MWA was successfully applied in the conservative management of a heterotopic cervical pregnancy in the first trimester. The procedure was easy to perform and was well tolerated and, most importantly, with no consequences on intrauterine pregnancy. This technique through different kind of intra-procedural controls permitted to obtain a more predictable spherical ablation volume, based on the position of the antenna, allowing the operator to safely keep the margins of the ablation zone within the gestational sac and to preserve the integrity of the cervix. Even if the termination of the pregnancy was immediate, with no ultrasound visualization of both the gestational sac and the embryo, the ultrasound showed throughout the pregnancy the presence in the cervical canal of nonhomogeneous vascularized material whose dimensions halved with time, without ever disappearing completely. However, the cervical length was always normal and funneling absent. The presence of this material was also the cause of the bleeding that occurred at the end of pregnancy as soon as the first uterine contractions occurred, fortunately without important consequences.

In summary, our case shows that heterotopic cervical pregnancies can be successfully and conservatively treated with MWA during the first trimester.

## Electronic supplementary material

ESM 1 (MP4 20,565 kb)
